# Using satellite‐derived estimates of plant phenological rhythms to predict sage‐grouse nesting chronology

**DOI:** 10.1002/ece3.6758

**Published:** 2020-09-15

**Authors:** David C. Stoner, Terry A. Messmer, Randy T. Larsen, Shandra Nicki Frey, Michel T. Kohl, Eric T. Thacker, David K. Dahlgren

**Affiliations:** ^1^ Department of Wildland Resources Utah State University Logan UT USA; ^2^ Department of Plant and Wildlife Sciences Brigham Young University Provo UT USA; ^3^ Warnell School of Forestry and Natural Resources University of Georgia Athens GA USA

**Keywords:** green wave, nest initiation, Normalized Difference Vegetation Index, phenology, sage‐grouse, satellite imagery

## Abstract

The “green wave” hypothesis posits that during spring consumers track spatial gradients in emergent vegetation and associated foraging opportunities. This idea has largely been invoked to explain animal migration patterns, yet the general phenomenon underlies trends in vertebrate reproductive chronology as well. We evaluated the utility of this hypothesis for predicting spatial variation in nest initiation of greater sage‐grouse (*Centrocerus urophasianus*), a species of conservation concern in western North America. We used the Normalized Difference Vegetation Index (NDVI) to map the green wave across elevation and then compiled dates and locations of >450 sage‐grouse nests from 20 study sites (2000–2014) to model nest initiation as a function of the start of the growing season (SOS), defined here as the maximum daily rate of increase in NDVI. Individual sites were drawn from three ecoregions, distributed over 4.5° latitude, and spanning 2,300 m in elevation, which captured the climatic, edaphic, and floristic diversity of sagebrush ecosystems in the southern half of current sage‐grouse range. As predicted, SOS displayed a significant, positive relationship with elevation, occurring 1.3 days later for each 100 m increase in elevation. In turn, sage‐grouse nest initiation followed SOS by 22 ± 10 days (*r^2^* = .57), with hatch dates falling on or just prior to the peak of the growing season. By timing nesting to the green wave, sage‐grouse chicks hatched when the abundance of protein‐rich invertebrate biomass is hypothesized to be nearing a seasonal high. This adaptation likely represents a strategy for maximizing reproductive success in the arid, variable environments that define sagebrush ecosystems. Given projected changes in climate and land use, these results can be used to predict periods of relative sensitivity to habitat disturbance for sage‐grouse. Moreover, the near real‐time availability of satellite imagery offers a heretofore underutilized means of mapping the green wave, planning habitat restoration, and monitoring range conditions.

## Graphical abstract

We tested the hypothesis that sage‐grouse nesting chronology would correspond to the “green wave.” Sage‐grouse nest initiation tracked the start of the growing season (the “green wave”) by 22 ± 10 days, with hatch dates falling on or just prior to the peak of the growing season. This adaptation likely represents a strategy for maximizing reproductive success in the arid and variable environments that define sagebrush ecosystems.

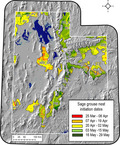

## 1 INTRODUCTION

In temperate latitudes, the vernal period of rapid vegetative growth corresponding to warming temperatures has become known as the “green wave” (van der Graaf, Stahl, Klimkowska, Bakker, & Drent, 2006; Van Wijk et al., 2011). Phenological development of the plant community follows temperature gradients correlated with latitude and elevation. This progression begins with grasses and proceeds to forbs, shrubs, and finally deciduous trees. For consumers, herbaceous vegetation is most palatable during early growth phases between the start and peak of the growing season. As plants wither and cell walls harden, herbage becomes less digestible and therefore of reduced caloric value (Bell, 1971; Sparks, Crick, Dunn, & Sokolov, 2013). The emergence of invertebrates closely follows flowering, and abundance wanes with botanical desiccation (Forrest & Thomson, 2011; Losey & Vaughn, 2006). In response to these patterns, consumers optimize fitness by synchronizing energetically taxing life stages to this predictable pulse of resources (Duursma, Gallagher, & Griffith, 2019; Martin, 1987). The ecological importance of this phenomenon suggests that mapping phenological rhythms may help inform conservation strategies for sensitive species in multiple‐use landscapes.

Parturition and early juvenile rearing periods are often the most energetically demanding phases of vertebrate life history, such that the timing of reproduction is under strong selection pressure (Blomberg, Gibson, Atamian, & Sedinger, 2017; Martin, 1987). The green wave hypothesis has been used to explain habitat selection and migration patterns of various avian and mammalian species (Avgar, Mosser, Brown, & Fryxell, 2013; Bischof et al., 2012; Drent, Ebbinge, & Weijand, 1978; van der Graaf et al., 2006; Shariatinajafabadi et al.., 2014) and has potential for predicting reproductive patterns as well (Kerby & Post, 2013). By timing parturition to the green wave, individuals can maximize reproductive success through enhanced nutrition. For example, mule deer (*Odocoileus hemionus*) synchronize birthing to a window between the start and peak of the growing season, with highest fawn mortality coinciding with weak or unpredictable growing seasons (Stoner, Sexton, Nagol, Bernales, & Edwards, 2016). Several hypotheses have been put forth to explain avian reproductive chronology, including photo‐period, female energetics, seasonal development of reproductive organs, and phenology of important food resources (Dunn, 2004). Although mechanisms are still unclear, all are correlated with temperature. Given the hypothesized link between the timing of nest initiation and juvenile survival with food abundance (Blomberg, Poulson, Sedinger, & Gibson, 2013; Dunn, 2004), the ability to predict plant and animal phenology from satellite‐based measures of the green wave may be of value for avian conservation (Cole, Long, Zelazowski, Szulkin, & Sheldon, 2015; Smith, Steenhof, McClure, & Heath, 2017).

**FIGURE 1:**
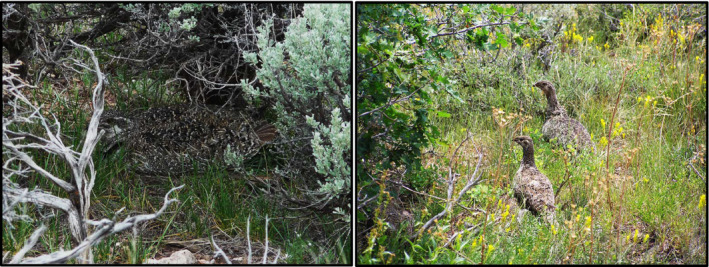
Sage‐grouse use camouflage and vegetative cover to maximize nest success. Following hatch, females and chicks require a protein‐rich diet dominated by forbs and invertebrates (Diamond Mountain, Utah; photos courtesy of Josh Kaze, Brigham Young University)

The greater sage‐grouse (*Centrocercus urophasianus;* hereafter sage‐grouse) is a widely distributed, ground‐dwelling game bird indigenous to western North America (Figure 1). As the name implies, the species is associated with sagebrush (*Artemisia* spp.) ecosystems, which occur over a wide range of latitudes, elevations, and climatic regimes. Sage‐grouse have declined in extent and abundance over the last 60 years primarily as a result of anthropogenically driven loss and fragmentation of sagebrush habitats (Knick et al., 2003). In response to these trends, sage‐grouse were identified as a candidate species for protection under the Endangered Species Act (ESA; U.S. Fish and Wildlife Service [USFWS] 2010) in the early 2000s. However, in 2015, due to unprecedented collaborative efforts by multiple public and private jurisdictions across sage‐grouse range, the USFWS determined that sage‐grouse conservation threats had been mitigated and the species did not warrant ESA protection (USFWS, 2015). In the wake of the 2015 listing decision, joint endeavors among public and private stakeholders have continued to address range‐wide conservation threats, albeit on reduced budgets (Stiver et al., 2015). Maintaining these efforts over large expanses of habitat will require continued monitoring of conservation actions to document the effects of habitat restoration on population change, and to parameterize the timing of life stages sensitive to disturbance, such as nesting (Dahlgren, Guttery, et al., 2016).

Sage‐grouse management interventions typically attempt to improve population viability by influencing vital rates through legal protections, habitat restoration, and predator control (Taylor, Walker, Naugle, & Mills, 2012). In multiple‐use landscapes, protective management efforts may include adjusting the timing of temporary but potentially conflicting land‐use activities, such as prescribed burns, range manipulations, or recreational/industrial uses. Land managers use standards derived from the literature or synthesized guidelines to direct conservation efforts for species of concern. Contemporary sage‐grouse conservation strategies have largely focused on maintaining microhabitat conditions correlated with vital rates such as nest success, brood success, and female survival (Dahlgren, Guttery, et al., 2016; Stiver et al., 2015). However, these guidelines were developed using the limited data available at the time, which did not encompass the full range of variability defining sage‐grouse habitat (Boyd, Johnson, Kerby, Svejcar, & Davies, 2014; Connelly, Schroeder, Sands, & Braun, 2000; Dahlgren et al., 2019; Hagen, Connelly, & Schroeder, 2007). To improve the efficacy of interventions and inform land‐use planning, natural resource managers require models predicting the timing of sensitive life stages over the range of climatic and environmental conditions under which sage‐grouse occur. Given the costs of assessing habitat at statewide or national scales, the ready availability of near real‐time satellite imagery may offer an economical and consistent means of synoptically monitoring habitat conditions. By tracking phenological variation across latitude and elevation, practitioners can tailor management policies to local conditions. For example, avoiding mismatches between animal and plant phenology by translocating birds within common climatic‐phenological contours during population augmentation or reintroductions.

Reports from the literature suggest a wide range of variation in the timing of lekking (breeding) and nest initiation (Schroeder, Young, & Braun, 1999), but despite the importance of this life history trait (Dahlgren, Guttery, et al., 2016; Taylor et al., 2012), there have been no systematic inquiries into the climatic conditions that underlie the timing of sage‐grouse reproductive schedules. The onset of the green wave is climatically determined and associated with a predictable pulse of forb and invertebrate biomass. Given the asynchrony in nesting dates observed among sage‐grouse populations (Connelly, Rinkes, & Braun, 2011), we hypothesized that within a given population, sage‐grouse nesting chronology would be timed to the arrival of the green wave (Dunn, 2004). To test this hypothesis, we combined satellite‐derived indices of primary production with telemetry‐based field data collected from 20 study sites distributed across three ecoregions. To the extent that reproductive events correspond to the green wave, the ability to map the timing of this phenological phenomenon can enhance species conservation planning in multiple‐use landscapes.

## 2 METHODS

### 2.1 Study region

**FIGURE 2:**
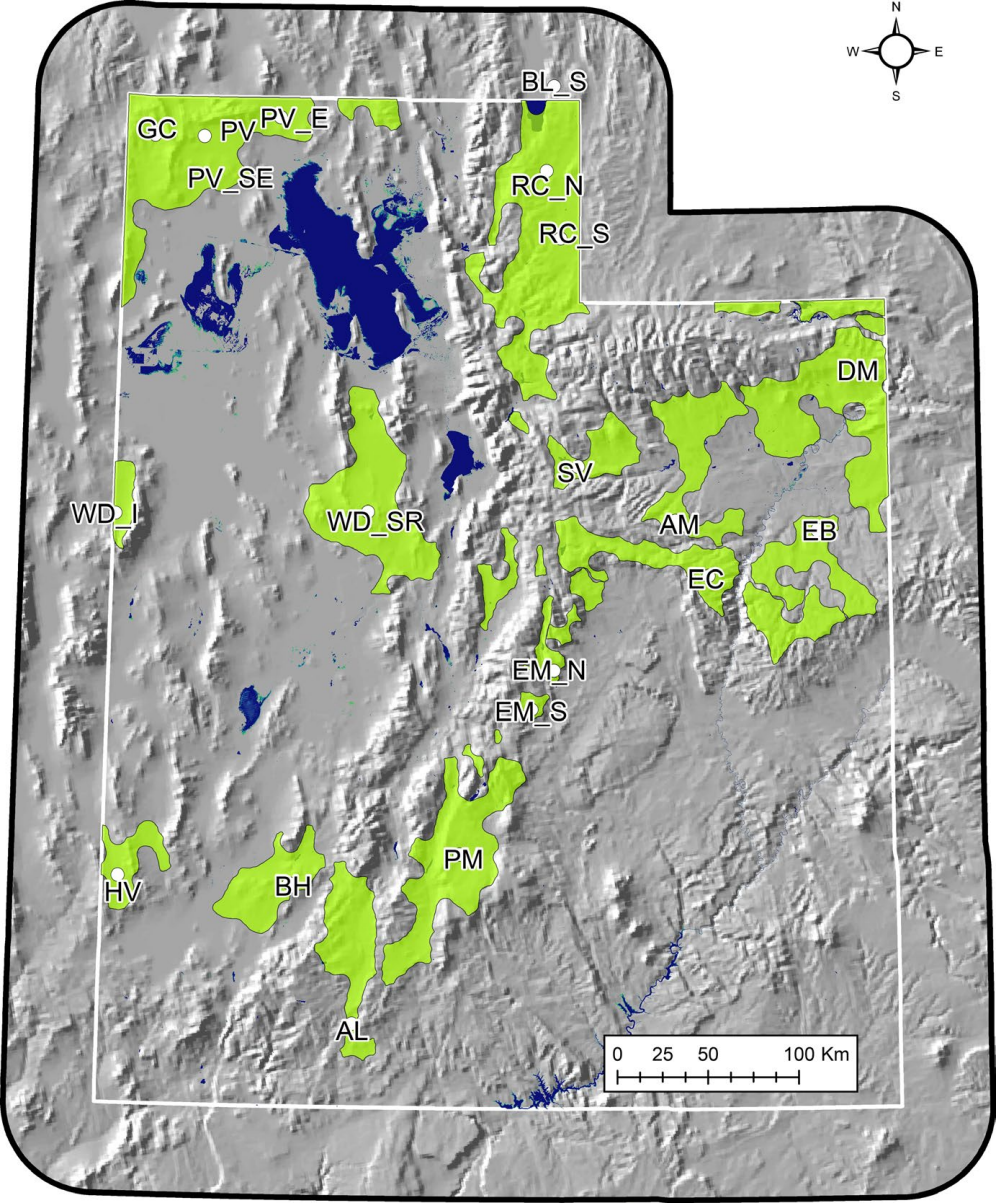
Green polygons represent currently occupied greater sage‐grouse habitat in Utah; white dots represent study site centroids. Nest initiation data were collected on 20 sites within three ecoregions (Table 1), representing a range of floristic, edaphic, and climatic conditions (2000–2014)

To evaluate the green wave hypothesis, we compiled extant datasets of sage‐grouse nest locations and dates sampled from the largest remaining sagebrush habitats within the Utah portions of the Great Basin, Colorado Plateau, and Wyoming Basins ecoregions (Dahlgren et al., 2015). Historically, sage‐grouse were distributed throughout sagebrush‐dominated ecosystems in Utah (~73,000 km^2^), but current estimates indicate they are absent from almost 60% of their historic statewide range (~29,800 km^2^; Beck, Mitchell, & Maxfield, 2003). The largest remaining populations inhabit Grouse Creek/Park Valley in the northwest, Diamond Mountain in northeastern Utah, the east side of the Bear River Mountains in north‐central Utah (Rich County, Bear Lake), and Parker Mountain in south‐central Utah (Figure 2, Table 1). Smaller populations are found throughout remaining sagebrush‐dominated sites.

**TABLE 1:** Geographic and phenological characteristics for sage‐grouse study sites in Utah, 2000–2014

Site	Name	LAT	LONG	Elev (m)	Growing Season Julian Dates						Length of Season (in days)		NESTS (*n*)
					Start		Peak		End				
					Mean	*SD*	Mean	*SD*	Mean	*SD*	Mean	*SD*	
AL	Alton Valley	37.43	−112.46	2,108	94	36	208	27	315	32	221	51	8
AM	Anthro Mtn	39.92	−110.43	2,543	100	17	204	16	319	18	219	22	108
BH	Bald Hills	38.11	−112.87	2,203	88	25	175	18	274	31	187	28	12
BL_S	Bear Lake, South	42.07	−111.21	2,129	97	16	183	10	317	17	220	19	22
DM	Diamond Mtn	40.65	−109.22	2,260	103	22	187	28	308	23	217	51	88
EB	East Bench	39.86	−109.52	1,745	80	37	176	27	321	45	268	75	3*
EC	East Carbon	39.64	−110.21	2,579	94	29	200	21	323	21	235	38	3*
EM_N	Emery, North	39.18	−111.20	2,563	85	24	199	20	334	13	250	28	11
EM_S	Emery, South	38.98	−111.35	2,578	88	24	201	18	329	14	242	32	13
GC	Grouse Creek	41.78	−113.86	1,877	90	13	190	15	314	24	224	20	80
HV	Hamlin Valley	38.13	−113.95	1,967	75	27	174	30	283	52	224	81	6*
PM	Parker Mtn	38.26	−111.80	2,669	88	21	209	17	328	9	239	25	218
PV	Park Valley	41.79	−113.55	1,916	94	16	181	17	284	35	191	30	72
PV_E	Park Valley, East	41.90	−113.03	1,372	74	33	165	25	289	50	219	82	4
PV_SE	Park Valley, Southeast	41.63	−113.37	1,602	74	22	176	34	277	41	226	78	5
RC_N	Rich County, North	41.66	−111.26	2,071	93	17	173	14	298	30	205	21	38
RC_S	Rich County, South	41.32	−111.17	2,093	95	14	179	14	303	32	208	26	25
SV	Strawberry Valley	40.19	−111.04	2,348	108	14	192	8	317	9	209	13	202
WD_I	West Desert, Ibapah	39.92	−114.05	1,802	93	41	174	27	274	38	192	74	9
WD_SR	West Desert, Sheeprock	39.96	−112.42	2,041	101	27	178	23	275	37	178	46	30

Note

Measures were derived by buffering each nest location, regardless of fate, by 500 m and dissolving all nests into a common polygon by study site. Estimates of phenological dates represent the mean for the entire polygon across years. Asterisks in the “NEST” column indicate all nests failed and were therefore excluded from nest initiation date analyses.

Utah lies at the center of the Intermountain West, with sage‐grouse distribution in this region straddling a transition zone between sagebrush‐steppe and sagebrush semi‐desert vegetative associations (West, 1983). Both are shrub‐dominated communities, but variation in the form, timing, and amount of precipitation influences the floral composition of the understory. On snow‐dominated sites in northern Utah, sagebrush understories contain a higher proportion of herbaceous vegetation cover than the monsoon‐influenced semi‐desert sites in the southern part of the study region. Generally, big sagebrush (*A. tridentata*) is dominant on most occupied sites, with Wyoming (*A. t. wyomingensis*), basin (*A. t. tridentata*), and mountain (*A. t. vaseyana*) big sagebrush at lower, mid, and high elevations, respectively. Shallower soils support communities dominated by low (*A. arbuscula*) and black (*A. nova*) varieties of sagebrush. High elevation and mesic sites throughout the region contain silver sagebrush (*A. cana*), and small, local populations of three‐tip sagebrush (*A. tripartite;* Schultz, 2012) occur in the northern portions of the state.

To sample the variation in environmental conditions across the region, we compiled sage‐grouse nest location data from 20 individual study sites collected between 1998 and 2014 (Figure 2, Table 1). Collectively, study sites covered 4.5° latitude and over 2,300 m in elevation, thereby capturing the broad range of climatic, edaphic, and taxonomic diversity of sagebrush ecosystems in the southern half of current sage‐grouse range (Dahlgren, Messmer, et al., 2016).

### 2.2 Nest Initiation dates

Sage‐grouse exhibit a precise reproductive schedule that begins with lekking in early spring, followed by initiation (sequential laying of eggs), incubation (hens restricted to the nest), and finally the hatch (Schroeder et al., 1999). To evaluate the role of plant phenology in this process we captured female sage‐grouse on or near lek sites during the breeding season (~March‐April; Dahlgren et al., 2019), prior to nest initiation. Following established protocols, we conducted captures at night using all‐terrain vehicles, spotlights, and dip nets (Connelly, Reese, & Schroeder, 2003; Giesen, Schoenberg, & Braun, 1982; Wakkinen, Reese, & Connelly, 1992). All birds were outfitted with 22 g necklace style very‐high frequency (VHF) radio transmitters (Advanced Telemetry Systems, Isanti, MN, USA, and Holohil Systems, Ltd., Carp, Ontario, Canada). On some sites, birds were also marked with a numbered aluminum leg band (National Band Company, Newport, KY). During capture, we recorded sex, age, body mass, behavior during handling, processing time, dominant vegetation, and GPS coordinates of the capture location (Universal Transverse Mercator [UTM], NAD 1983, Zone 12N; Crunden, 1963). All birds were processed on site and released immediately postprocessing to reduce the potential for stress‐related mortality. All capture and handling procedures were reviewed and approved by IACUCs from Utah State University (nos. 945‐R, 2322, 2411, 2419, 2560, 1451, 2189, 942, 942‐R, 1194, 1404, and 1332; Dahlgren, Messmer, & Koons, 2010), and Brigham Young University (nos. 100302, 110301, 050301, and 080402; Baxter, Baxter, Dahlgren, & Larsen, 2017).

Nest initiation refers to the multi‐day period in which a female grouse lays a clutch of eggs in a nest bowl. This phase typically occurs within three weeks of copulation (Knick & Connelly, 2011) and is characterized by short, local movements around the nest site. Mean clutch size is just over six eggs, laid at a rate of ~1.5 days/egg (Connelly et al., 2011; Giesen et al., 1982; Schroeder et al., 1999). Incubation begins once the entire clutch is laid. During this time, a female will sit on the nest for approximately 23 hr/day to regulate egg temperature and therefore chick development (Coates & Delehanty, 2008). The hatch is synchronized for all eggs and occurs after ~27 days of incubation (Schroeder, 1997). To document incubation, we used binoculars to observe radio‐marked females at daily to semi‐weekly intervals during the nest initiation period. We considered incubation to have commenced once we observed a given female on a nest during two consecutive visits. After confirmation, monitoring was increased to one visit every 2 to 3 days and then daily as the estimated hatch date approached. Nests were monitored until fate could be determined through visual inspection of the nest bowl, and observations of radio‐marked females with or without chicks following nest abandonment. Nests were considered successful if ≥1 egg hatched (Dahlgren et al., 2010).

Hatch dates were accurate to within 2 days and were the most precisely measured dates associated with the reproductive schedule. To estimate the dates of nest initiation, we back‐calculated from the hatch date assuming a mean of 27 days for incubation, plus 9 (the mean number of eggs in a clutch multiplied by 1.5 days/egg; Dahlgren et al., 2010). This produced a correction of −36 days from hatch for an estimate of the earliest nest initiation date.

Total sample size was 1,028 nests, but to match nest dates with available contemporaneous satellite data (see below), we excluded observations collected prior to 2000 (*n* = 40) for a sample of 988 nest locations. For analyses of nest timing, we excluded all failed nests because we did not have a hatch date from which to back calculate initiation. From the sample of 988, we were able to estimate hatch date for 460 nests. Sample size for individual study sites was highly variable, ranging from 3–218 nest locations/ site (mean ± *SD* =46 ± 63) and 1–137 successful nests/ site (mean ± *SD* =27 ± 39). Dates were converted to Julian (i.e., 1–365) and averaged across years by study site.

### 2.3 Environmental variables

Elevation. The study region is characterized by extensive topographic relief, which is strongly correlated with variation in temperature and precipitation (Banner, Baldwin, & Leydsman‐McGinty, 2009), making elevation a good index of local climate. We used a 90 m Digital Elevation Model hosted by the Utah Automated Geographic Reference Center to estimate mean elevations for all sage‐grouse nest locations and study sites (https://gis.utah.gov/data/indices/usgs‐dem‐indices/).

Temperature. Plant dormancy, germination, and leaf‐out are sensitive to soil temperature (Footitt, Douterelo‐Soler, Clay, & Finch‐Savage, 2011). This metric was not readily available, so to index growing season conditions we used air temperature data from the PRISM Climate Group (Oregon State University, http://prism.oregonstate.edu). Daily temperature means were downloaded for the months March‐June (2000–2014) at 4‐km spatial resolution. From these data, we estimated soil thaw date for each study site, defined here as the date between 1 March and 1 July after which mean daily air temperatures consistently exceeded 0°C, that is, (daytime high + daytime low)/2 > 0.

Plant phenology. The satellite‐derived Normalized Difference Vegetation Index (NDVI; Tucker, 1979) is a widely used means of measuring plant phenology synoptically. NDVI is an index of photosynthetic cover, scaled from −1 to 1. It has been used extensively to index forage quality for ungulates (Garroutte, Hansen, & Lawrence, 2016), track avian migration (van der Graaf et al., 2006), model avian behavioral responses to phenological changes in vegetation (Kelly et al., 2016; Smith et al., 2017; Thorup et al., 2017), predict insect emergence and abundance (Cole et al., 2015; Lassau & Hochuli, 2008), and to map plant phenology across climatic regimes (Stoner et al., 2016). Here we use NDVI to quantify plant phenological patterns for each nest location and study site, and to serve as an index of herbaceous plant cover and invertebrate biomass.

To achieve this, we used daily, 500 m resolution images of surface reflectance from the Moderate‐Resolution Imaging Spectroradiometer (MODIS) sensors aboard the Terra and Aqua satellites. Data were compiled from 2000–2014 and masked for cloud and snow cover, corrected for solar angle illumination effects (i.e., Bidirectional Reflectance Distribution Function), and smoothed using a locally weighted scatterplot smoothing function (LOWESS). From these data, we calculated Julian dates for the start (SOS), peak (POS), and end (EOS) of the growing season, along with their associated NDVI values. SOS and EOS were defined as the inflection points on the ascending and descending arms of the seasonal growth curve, respectively. POS was the date on which the highest value of NDVI was recorded during the growing season. In sagebrush‐dominated communities, multiple peaks within a growing season are common. These stem from the late spring/early summer flush associated with the herbaceous understory and the emergence of annual leaves on sagebrush plants. The late summer/early fall peaks represent sagebrush flowering events. Here, we used the highest peak recorded during a given season, regardless of phenophase, which is typically the first. Length of season (LOS) was estimated as the time between EOS and SOS, averaged across years. Procedures related to NDVI data development are detailed in Stoner et al. (2016) and Nagol, Sexton, Anand, Sahajpal, and Edwards (2017).

In some parts of the northern hemisphere, the start of spring is advancing with increasing temperatures (Karkauskaite, Tagesson, & Fensholt, 2017; Wang et al., 2015). Because this could act as a confounding factor, we conducted linear regressions on each study site and a statewide assessment (pooled sites) over an 18‐year interval (2000–2017) to identify any trends in the timing of SOS or POS.

### 2.4 Sampling frame

We sampled elevation and phenological metrics at the scale of the study site (>1 km^2^), and the nest location (250 m radius or ~0.25 km^2^). Study sites encompassed a variety of land uses and associated plant communities, many of which are not suitable sage‐grouse habitat. To adequately sample study sites while minimizing the influence of unused landcover types, we buffered each nest by 500 m to create a ~0.75 km^2^ polygon around each nest. This was done for all nests, regardless of fate (*n* = 988). We then dissolved the buffered nest locations by study site to create a master sampling polygon for a given study site. The size of resulting polygons varied with the number and distribution of nests (range: 3–134 km^2^; mean ± *SD* = 22 ± 31 km^2^). Because PRISM temperature data were only available at a 4‐km resolution, those data were sampled solely at the scale of the study site using the buffered polygons.

Incubating sage‐grouse typically forage within a ~0.20–1 km^2^ radius of the nest (Schroeder et al., 1999), which approximated the size of a single NDVI pixel. Thus, we used UTM coordinates of the nest location and nest year for all successful nests to sample concurrent NDVI. This effectively captured the plant phenology individual birds were exposed to during a year‐specific reproductive event.

### 2.5 Analytical techniques

To test our hypothesis we conducted separate analyses for each scale of investigation. At the scale of the study site, we examined variation in plant phenology as a function of elevation and associated temperature relationships. This provided a description of the physical/climatic differences between the sage‐grouse study sites. At the scale of the nest site, we modeled nest initiation as a function of plant phenology, which served as a formal test of our working hypothesis.

#### 2.5.1 Model 1. Study site scale: elevation as a determinant of phenological events

Given the strong correlation between elevation and climate, we hypothesized that spring green‐up would occur later at higher elevations. To evaluate this, we used standard univariate linear regression techniques to create models predicting multiannual means in the timing of soil thaw, SOS, POS, EOS, and LOS as a function of elevation (m) for the interval 2000–2014. We also modeled interannual variation in the timing of each phenological date across elevation using the *SD* associated with SOS, POS, and EOS.

#### 2.5.2 Model 2. Nest site scale: testing the “green wave” effect on nest initiation dates

To model the relationship between sage‐grouse nest initiation and plant phenology we regressed nest dates directly on the concurrent SOS date for the nest pixel. We used this model to derive a mean lag time between spring green‐up and nest initiation. The final dataset was comprised of the estimated initiation date and associated SOS date for the specific location (pixel) of an individual successful nest. We weighted the regression model by sample size to compensate for the skewed distribution of nests/ study site. Dates for each variable were then averaged across years within study sites, so that each datum in the regression model represented mean nest and SOS dates for a given study site (2000–2014). The model predicted the Julian date of nest initiation as a function of SOS at the scale of the nest site (0.25 km^2^).

Lags between response variables measured in units of Julian dates were calculated by subtracting the earlier elevation‐based regression (e.g., SOS) from the mean of the later (e.g., POS). The range is reported as the low and high regression estimates, and variance was calculated as the *SD* of the regression estimates for each measured elevation value. All descriptive statistics are presented as the mean ± *SD* unless otherwise noted. For regressions, we evaluated statistical assumptions both formally (Shapiro–Wilk test) and visually through qqnorm plots of model residuals. All analyses were conducted in program R (R Development Core Team, 2013), and maps were produced using ArcGIS software (ESRI).

## 3 RESULTS

Sites demonstrated interannual variation in the timing of important phenological events, but we found no evidence for systematic trends in advancing SOS or POS. The resulting *r* ^2^ values ranged from .00 to .11 for SOS, and from .00 to .10 for POS. None of the regression slopes (for individual sites or for the statewide model) displayed statistically significant trends in either metric over an 18‐year interval that encompassed all data presented here (Appendix S1).

### 3.1 Model 1. Study site scale: elevation as a determinant of phenological events

**FIGURE 3:**
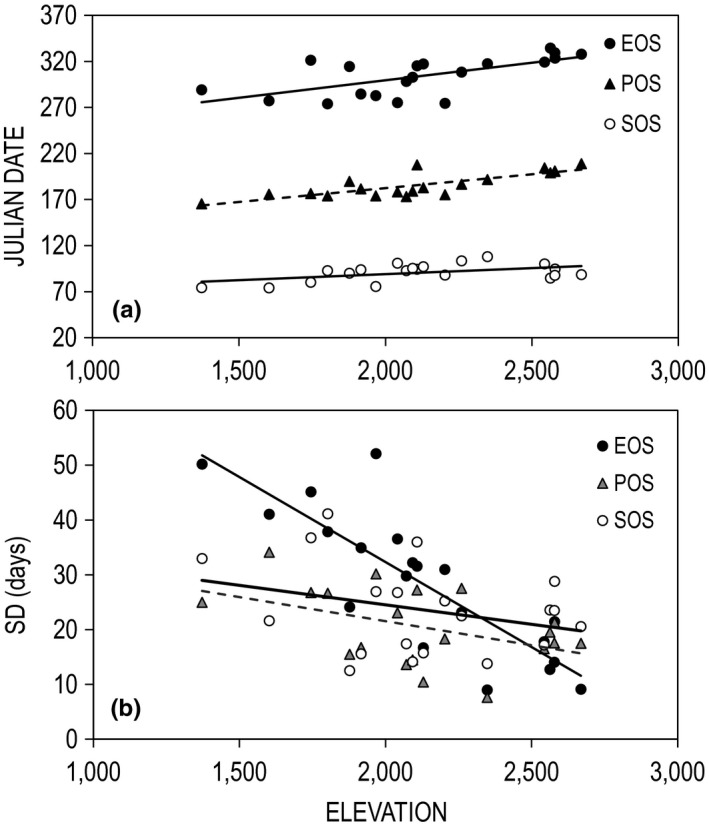
Panel A: predicted dates for start‐of‐season (SOS), peak‐of‐season (POS), and end‐of‐season (EOS) as a function of elevation (in m). Panel B: relationships between the variance in phenological dates (*SD* measured in days) and elevation. All data represent sage‐grouse study sites in Utah (*n* = 20; 2000–2014)

Dates of important climatic and phenological events varied positively with elevation (Figure 3a). The mean date on which daily air temperatures exceeded freezing (Thaw) ranged from 5 March to 4 April (22 March ± 9 days). Start‐of‐season (SOS) varied from 15 March‐18 April (1 April ± 12 days). In turn, POS and EOS ranged from 14 June to 27 July (5 July ± 13 days), and 1 October to 30 November (31 October ± 21 days), respectively. Length of the growing season ranged from 178–268 days (219 ± 22 days; Table 1).

Using elevation as a common predictor, model results indicated that SOS tracked mean thaw date by 5–14 days (9 ± 2 days), and POS followed SOS by 83–105 days (95 ± 6 days). Thaw date, SOS, and POS occurred 2, 1.3, and 3 days later for each 100 m increase in elevation, respectively. Length of the growing season increased by 4 days for each 100 m increase in elevation. All responses showed significant relationships with elevation (Table 2).

**TABLE 2:** Regression models predicting the Julian dates for thaw, start, peak, end, and length of the growing season as a function of elevation in Utah (2000–2014; *n* = 20)

Factor	*F*	*df*	*p*	Model	*r* ^2^
Thaw date	26.9	1, 18	<.0001	*y* = 0.02*x* + 39.2	.60
Start of season	5.5	1, 18	.03	*y* = 0.013*x* + 63.1	.23
Peak of season	32.1	1, 18	<.0001	*y* = 0.03*x* + 122.5	.64
End of season	13.2	1, 18	.00	*y* = 0.04*x* + 223.9	.42
Length of season	9.9	1, 18	.005	*y* = 0.04*x* + 127.2	.36

Note

All phenological variables exhibited a significant relationship with elevation.

Precision in phenological dates increased with elevation; that is, the interannual variance in SOS, POS, and EOS was smaller at higher elevations. Using the *SD* in phenological dates as an index of interannual variability, SOS, POS, and EOS were all negatively correlated with elevation (Figure 3b). The *SD* in POS (*F* = 4.6; *df* = 1, 18; *p* = .05) and EOS (*F* = 42.9; *df* = 1, 18; *p* < .01) exhibited significant relationships with elevation but SOS did not (*F* = 1.8; *df* = 1, 18; *p* = .19). The *SD* in POS ranged from 8–34 days (mean = 20 days), whereas EOS ranged from 9–66 days (mean = 29 days). For each 100 m increase in elevation, the *SD* in the onset of POS and EOS were reduced by 1 and 3 days, respectively. Variation in LOS was also significant (*F* = 4.9; *df* = 1, 18; *p* = .005), stemming from the strong effect of EOS (*SD*). Counterintuitively, LOS was greater at higher elevations.

### 3.2 Model 2. Nest site scale: testing the “green wave” effect on nest initiation dates

**FIGURE 4:**
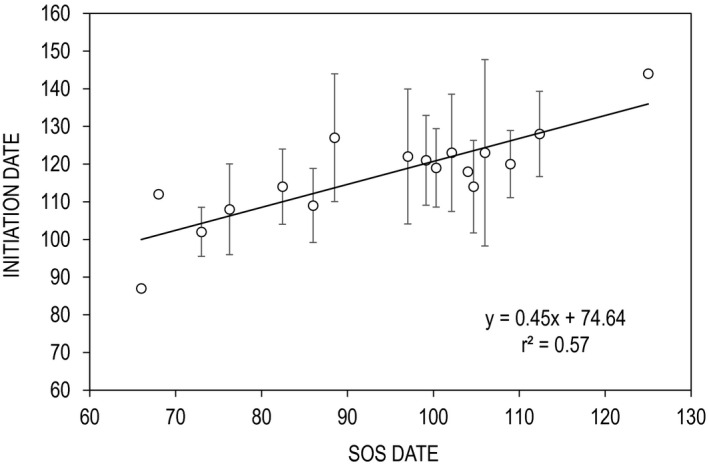
Mean Julian dates for sage‐grouse nest initiation, by study site, as a function of mean start‐of‐season (SOS) dates (2000–2014; *n* = 17). On average, sage‐grouse initiate nesting 22 (±10) days after SOS. Error bars represent interannual variation in nest dates, by site. Points without bars indicate lack of variance due to small sample sizes. Three sites had no successful nests and were therefore censored from the analysis

**FIGURE 5:**
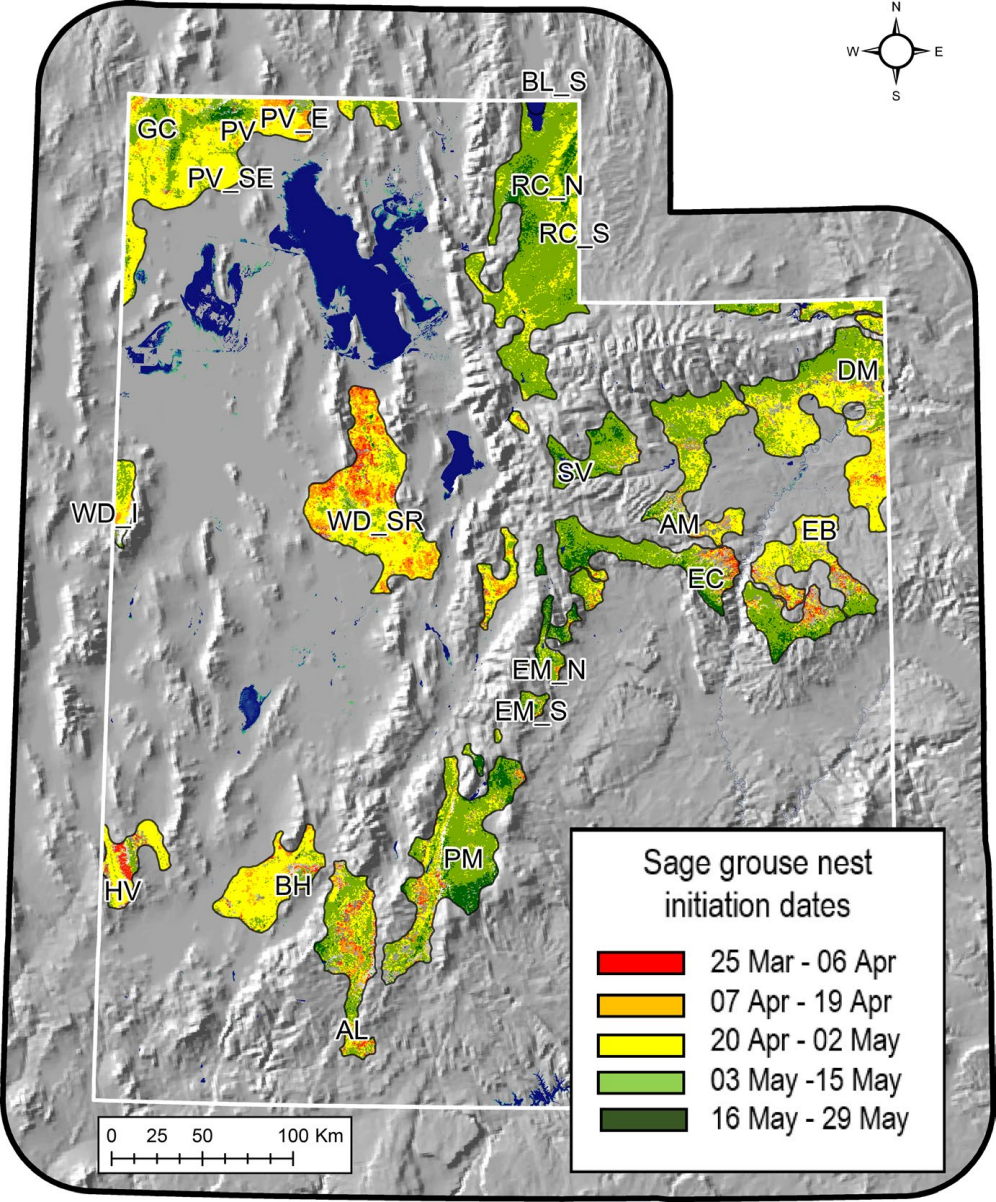
Predicted nest initiation dates for greater sage‐grouse in Utah. Map represents a spatially explicit version of the regression model in Figure 3, in which *x* = mean Julian date of SOS (2000–2014)

**FIGURE 6:**
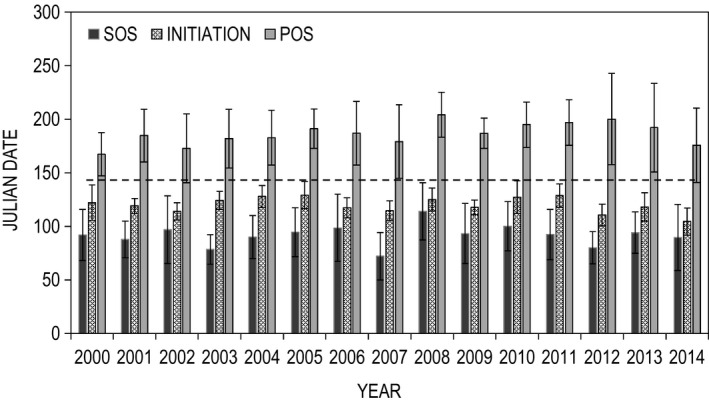
Mean statewide sage‐grouse nest initiation dates tend to fall between the start (SOS) and peak of the growing season (POS), annually. Error bars represent variance (*SD*) across sites within a given year. Dashed line represents the mean statewide hatch date across years

Nest initiation dates ranged from 28 March to 24 May (28 April ± 13 days), with hatch dates falling between 3 May and 29 June (3 June ± 13 days). Initiation dates were best explained by corresponding measures of SOS date (*F* = 20.2, *df* = 1, 15; *p* = .0004; Figure 4). Model estimates indicated that nest initiation followed SOS by ~22 ± 10 days, approximating the date on which mean daily air temperatures exceeded 7°C. Importantly, the lag between SOS and nest initiation became shorter as SOS occurred later in the season (Figure 4). Based on these estimates, mean hatch dates occurred approximately 2–3 weeks prior to POS. Spatial variation in nest initiation is illustrated in Figure 5, and mean statewide temporal variation is displayed in Figure 6. Notably, at the statewide scale the nesting period fell between SOS and POS for every year of the study.

## 4 DISCUSSION

Biogeographically, Utah sage‐grouse populations cover a wider elevational range than those in most other western states. Prevailing arid conditions support sagebrush‐dominated plant communities across >2,300 m in elevation, ranging from a low lying band around the Great Salt Lake at the bottom of the Great Basin (Sheeprock, Park Valley units), into the highlands of the Colorado Plateau (Parker Mountain; Figure 2, Table 1). As such, local growing season parameters vary widely between sites. Variation in plant phenology across spatial gradients has been termed the “green wave.” This phenomenon has been used to predict the timing of spring migration and reproductive schedules for numerous vertebrate species (Bischof et al., 2012; Thorup et al., 2017). Here, we evaluated the phenological rhythms of sagebrush communities, and its consequent effects on the nesting chronology of sage‐grouse, a species of management concern in western North America.

### 4.1 Elevation as a determinant of phenological events

Across sites, the start of the growing season was correlated with the date on which mean daily air temperatures exceeded 7°C. Coarse‐scale measures of air temperature proved a good indicator of the critical soil temperatures required for many cool‐season grasses and forbs to break winter dormancy (~5–7°C; Probert, 2000). Soil thaw triggered the start of the growing season and arrival of the green wave, which is marked by the emergence of grasses, forbs, and invertebrate hatches (Lassau & Hochuli, 2008). As predicted, this phenomenon occurred progressively later at higher elevations. Nearly all plant phenological dates (SOS, POS, EOS, and LOS) and their respective variances (*SD*) displayed statistically significant relationships with elevation. In mountain ecosystems, precipitation, temperature, and evapotranspiration are strongly correlated with elevation, which influence the timing, magnitude, and duration of the growing season. Given these patterns, elevation proved a simple, intuitive index of climatic differences among sagebrush habitats, which in turn had significant effects on sage‐grouse nesting chronology.

### 4.2 Testing the “green wave” effect on nest initiation dates

Dunn (2004) hypothesized that nest timing evolved in response to differential nest success with respect to food abundance. Our results largely support this argument. Within individual populations, the overall nesting season was highly variable. However, across space sage‐grouse timed nest initiation and subsequent incubation periods to the window between SOS and POS (Figures 3a and 6). Sage‐grouse initiated nesting approximately 22 days after SOS regardless of elevation (Figure 4), a time when forb and invertebrate abundance are increasing daily, suggesting that food production was the underlying factor influencing the nesting schedule. Similar results have been found among insectivorous passerines and raptors. For example, Cole et al. (2015) reported that emergence of caterpillars (*Operophtera* sp.) was correlated with the budburst of their host plant and that two *Parus* species timed hatch date to coincide with peak abundance of this prey item. Similarly, nesting dates of American kestrels (*Falco sparverius*) were correlated with the emergence of both insects and small mammals in agricultural landscapes (Smith et al., 2017). This phenomenon was also evident among migratory harriers (*Circus pygargus*) that tracked local grasshopper (*Acrididae* sp.) hatches in African desert systems (Trierweiler et al., 2013). Collectively, these results suggest avian nesting and migration patterns are timed to match local maxima in high protein food resources.

**FIGURE 7:**
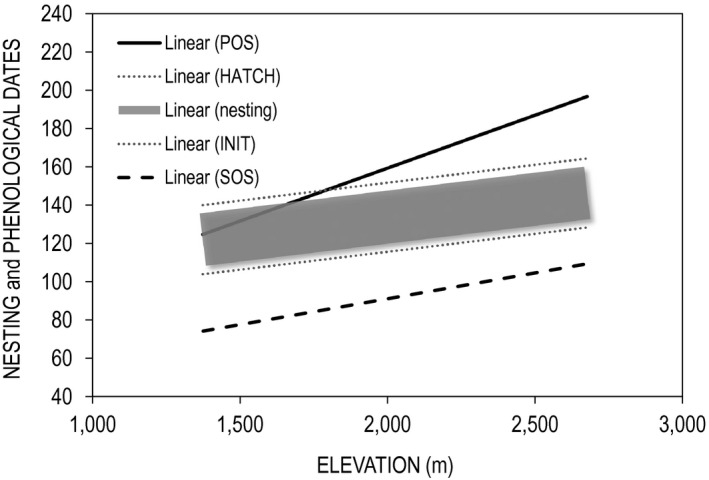
The nesting period, from initiation to hatch, as a shaded band between start‐of‐season SOS and POS for greater sage‐grouse study sites in Utah (2000–2014). Lines represent the fits of the linear models relating phenological and reproductive attributes with elevation

Food availability corresponds to season length, which varied as a function of plant desiccation at low elevations and frost at high elevations. Despite shorter frost‐free intervals at higher elevations, model results indicated that for each 100 m increase in elevation POS was delayed by 3 days and its corresponding variance reduced by 1 day. Similarly, LOS increased by 4 days for each 100 m increase in elevation, ranging from 182 days at 1,400 m, to 232 days at 2,700 m. Presumably, this pattern would reverse at some higher elevation, but within our sample the highest sites had the longest growing seasons. These patterns were influenced by the summer moisture regime. On high elevation sites, the growing season began with spring snowmelt but was extended by late summer monsoonal rains, resulting in a second growth pulse comprised of warm season grasses and forbs, thereby extending the period of food abundance (Wenninger & Inouye, 2008; Figure 7). Conversely, lower sites typically exhibited a single, short growing season that began in late spring, and was quickly followed by summer desiccation. Indeed, below 1,800 m sage‐grouse hatch dates tended to occur on, or just after, POS, whereas above this threshold, hatch occurred prior to POS (Figure 7). We suggest two complimentary explanations for this pattern.

First, reproductive success in sage‐grouse is associated with forb and invertebrate availability (Drut, Crawford, & Gregg, 1994). Winter diets of sage‐grouse consist almost entirely of sagebrush (Connelly et al., 2000; Crawford et al., 2004; Dahlgren et al., 2015). This is sufficient for the maintenance of basic metabolic needs, but to support egg development during the pre‐laying period, females require a diet high in protein, calcium, and phosphorus (Gregg, Dunbar, Crawford, & Pope, 2006). As defined here, SOS is not the first snow‐free day or sign of grass emergence, but the date on which NDVI reaches its maximum daily rate of change (Bischof et al., 2012; Nagol et al., 2017), and thus, the period of increasing herbaceous vegetation and arthropod availability (Forrest & Thomson, 2011). By initiating 3–4 weeks after SOS, females can diversify their diet, and obtain essential nutrients required for egg development and nesting activity (Barnett & Crawford, 1994; Gregg et al., 2006).

Second, and more striking, is the precision with which females timed initiation so that hatch occurred on or around POS. From a caloric standpoint, POS represents maximum ecosystem productivity, the period when frosts have ended, and forbs and arthropods are most abundant. Given that recruitment determines population growth (Dahlgren, Guttery, et al., 2016), we predicted that sage‐grouse would maximize chick survival by timing the hatch to a window corresponding to high food abundance. Based on model estimates, incubation begins approximately 43 days prior to POS and requires ~ 27 days to complete (Schroeder, 1997). This puts the mean hatch date approximately 16 days prior to POS—a period when food resources are increasing and diversifying on a daily basis.

To meet the protein and energetic demands required for growth, arthropods comprise 60%–80% of chick diets through the first three weeks posthatch (Dahlgren et al., 2015) and remain important up to 12 weeks posthatch (Blomberg et al., 2013; Gibson, Blomberg, Atamian, & Sedinger, 2017; Gregg & Crawford, 2009). During this period, sage‐grouse chicks are vulnerable to predation; accordingly, survival curves have the steepest declines during the first 14 days posthatch (Dahlgren et al., 2010; Gregg et al., 2006). Development of flight abilities is therefore critical to reduce mortality during this window of vulnerability. Chicks begin short flights as early as 14 days posthatch and can fly >100 m by 21 days posthatch. Our results demonstrate that across large landscapes with high levels of spatial heterogeneity in plant phenology, female sage‐grouse effectively synchronize energetic demands of newly hatched chicks to readily available sources of protein (Dahlgren et al., 2015). This timing insures chicks have the highest probability of obtaining resources needed to develop flight at the earliest possible age, which ultimately increases survival and hence, recruitment.

Sage‐grouse are indigenous to arid, drought‐prone environments defined by high interannual variability in water availability and growing season length (Blomberg et al., 2017). If sage‐grouse nest initiation is cued by SOS, then within‐population nest synchrony should reflect the degree of variability in the timing of SOS for a particular site. Given that long‐term forecasts for western North America suggest accelerating land‐use and climate changes in the coming decades (Garfin, Jardine, Merideth, Black, & LeRoy, 2013; Seager et al., 2012) we expect several patterns to emerge along elevational clines. First, within these dry habitats, sage‐grouse seek the relatively more productive sites during the brood‐rearing phase to extend access to succulent vegetation (Kane, Sedinger, Gibson, Blomberg, & Atamian, 2017). To mitigate plant desiccation birds can select wetland habitats (Donnelly et al., 2018; Donnelly, Naugle, Hagen, & Maestas, 2016), or in areas of high topographic relief, track succulent vegetation upslope or across aspects (Dahlgren, Messmer, et al., 2016). Importantly, year‐to‐year variability in the onset of phenological dates diminished with increasing elevation. That is, on high elevation sites POS occurred later and with greater predictability than on low sites. In conjunction with a longer growing season, this pattern allowed high elevation birds to nest well in advance of POS. In contrast, low‐elevation birds had shorter growing seasons and hatched in time with POS, which suggests these broods would exhibit greater movements following flight development to track resources. This puts high elevation populations at a distinct advantage having longer, more predictable growing seasons of higher overall productivity. Under these conditions, we would expect a high degree of within‐population nest synchrony and greater average nest success.

Phenological mismatches between environmental cues and consumer life history events occur when climatic changes or increased variability outpace a species’ ability to adapt to optimal environmental conditions (Burgess et al., 2018). Mismatches have been documented for several avian species (Burgess et al., 2018; Jones & Cresswell, 2010), and are considered a potential near‐term threat to recruitment during periods of rapid change. Thus, a second point is that low‐elevation sites displayed more inherent variation in SOS and LOS, suggesting that during years with short growing seasons (i.e., drought years), birds missing the narrow window of forage availability would be at greater risk of producing a hatch after critical food resources had desiccated. Across years, we would expect these populations to exhibit boom and bust dynamics in nest success, reflecting the erratic conditions under which they live. Similar patterns have been described for desert ungulates, in which a highly variable growing season promotes a long birthing season with only a small portion of any cohort surviving in a given year (Bunnell, 1982; Longshore, Lowery, & Cummings, 2016; Stoner et al., 2016). Low‐elevation populations are more vulnerable to poor recruitment, with future changes likely to exacerbate current trends. Thus, if sage‐grouse nest initiation is ultimately driven by temperature effects on plant phenology, then changes in phenological rhythms driven by variation in climate and/or expansion of invasive species (Blomberg, Sedinger, Atamian, & Nonne, 2012; Clinton et al., 2010) have important implications for sage‐grouse conservation (Boyte, Wylie, & Major, 2016).

### 4.3 Management implications

Several practical implications stem from these findings. First, plant phenological patterns vary widely within the continental distribution of sage‐grouse. The factors predicting nest chronology in sage‐grouse are climatically driven, and as such our results are not specific to the populations studied here, but can be used to estimate nesting patterns in other parts of the species’ range. Additionally, sage‐grouse responses to plant phenology are likely typical of insectivorous avian species native to sagebrush ecosystems. If so, then conservation measures designed around sage‐grouse life history patterns may benefit other species. Monitoring the phenological phases of herbaceous vegetation may be one way of standardizing and modeling estimates of nest initiation across the varied ecosystems in which sage‐grouse occur. Estimates can then be used to refine the time frames used when buffering sage‐grouse nesting activity from land‐use disturbances.

Second, translocations are a commonly employed management technique for re‐establishing or supplementing small populations. Long‐lived organisms may have time to acclimate to new conditions following translocations (e.g., *Cervus elaphus* in the southern hemisphere; Caughley, 1970), but sage‐grouse are relatively short‐lived (~1.5–5 years; Schroeder et al., 1999), with the typical female undergoing only 1–4 nesting seasons over her lifespan. Given the cost of reintroduction efforts and the small number of breeding cycles, the loss of even one nesting season can compromise the success of a translocation effort. Anthropogenic mismatch stemming from the movement of birds from one climatic regime to another can be minimized by matching source and target populations within similar phenological contours and concurrent nesting rhythms (Figure 5).

Lastly, development and implementation of sage‐grouse conservation strategies under projected climate and land‐use change scenarios will depend on a range‐wide system for monitoring changes in habitat. The near real‐time availability of satellite imagery offers a heretofore underutilized means of monitoring range conditions and planning habitat restoration either synoptically, or in remote areas or where field data are logistically difficult to obtain. By employing these large‐scale measures, practitioners can tailor management policies and actions to local conditions to better time lek counts, nest surveys, predator control, and habitat restoration efforts.

## CONFLICT OF INTERESTS

The authors declare that they have no competing interest related to this manuscript.

## AUTHOR CONTRIBUTION

**David C. Stoner:** Conceptualization (lead); Formal analysis (lead); Methodology (equal). **Terry A. Messmer:** Conceptualization (supporting); Data curation (equal); Funding acquisition (lead); Project administration (lead). **Randy T. Larsen:** Data curation (equal); Funding acquisition (supporting); Investigation (supporting); Methodology (supporting); Project administration (supporting). **Shandra Nicki Frey:** Data curation (supporting); Formal analysis (supporting); Funding acquisition (supporting); Investigation (supporting); Project administration (supporting). **Michel T. Kohl:** Conceptualization (supporting); Data curation (supporting); Formal analysis (supporting); Methodology (supporting). **Eric T. Thacker:** Conceptualization (supporting); Data curation (supporting); Investigation (supporting). **David K. Dahlgren:** Conceptualization (equal); Data curation (lead); Formal analysis (supporting); Investigation (lead).

## Supporting information

Appendix S1

## Data Availability

Elevation data used in this analysis are available through the Utah Automated Geographic Reference Center web portal (https://gis.utah.gov/data/elevation‐and‐terrain/); PRISM data used to calculate thaw date can be downloaded from the Oregon State University web site (http://www.prism.oregonstate.edu/). Summary phenological data derived from satellite imagery are provided in Table 1. Raw MODIS data used to calculate NDVI data can be downloaded from the US Geological Survey web site (https://lpdaac.usgs.gov/). In Utah, sage‐grouse are legally classified as a sensitive species. As such, nest location data are considered proprietary and protected under state law. Data are not publically available except through a Government Records Access and Management Act request (GRAMA), under provisions stipulated by the Utah Division of Wildlife Resources (https://wildlife.utah.gov/grama.html).
