# Supplementary material for: Using satellite‐derived estimates of plant phenological rhythms to predict sage‐grouse nesting chronology
Source: Ecol Evol. 2020 Sep 15;10(20):11169–82. doi: 10.1002/ece3.6758 (PMC7593141; doi:10.1002/ece3.6758)
Supplement: Supplementary file 1 — Appendix S1 [file ECE3-10-11169-s001.pdf]

SITE	ELEV	r^2 (SOS)	df	F	P-value	r^2 (POS)	df	F	P-value
AL	2,108	0.01	1, 16	0.12	0.74	0.01	1, 16	0.20	0.66
AM	2,543	0.06	1, 16	0.94	0.35	0.03	1, 16	0.46	0.51
BH	2,203	0.08	1, 16	1.48	0.24	0.04	1, 16	0.66	0.43
BL_S	2,129	0.00	1, 16	0.02	0.88	0.01	1, 16	0.13	0.72
DM	2,260	0.00	1, 16	0.04	0.85	0.00	1, 16	0.07	0.80
EB	1,745	0.07	1, 13	0.97	0.34	0.01	1, 16	0.24	0.63
EC	2,579	0.03	1, 16	0.46	0.51	0.02	1, 16	0.25	0.62
EM_N	2,563	0.05	1, 16	0.80	0.38	0.00	1, 16	0.03	0.87
EM_S	2,578	0.00	1, 16	0.01	0.92	0.00	1, 16	0.01	0.91
GC	1,877	0.00	1, 16	0.00	1.00	0.00	1, 16	0.03	0.87
HV	1,967	0.01	1, 15	0.09	0.77	0.04	1, 16	0.68	0.42
PM	2,669	0.00	1, 16	0.00	0.99	0.00	1, 16	0.00	0.97
PV	1,916	0.01	1, 16	0.10	0.75	0.01	1, 16	0.16	0.70
PV_E	1,372	0.06	1, 16	1.10	0.31	0.00	1, 16	0.04	0.85
PV_SE	1,602	0.02	1, 15	0.32	0.58	0.05	1, 16	0.92	0.35
RC_N	2,071	0.01	1, 16	0.12	0.74	0.01	1, 16	0.20	0.66
RC_S	2,093	0.00	1, 16	0.07	0.80	0.02	1, 16	0.28	0.60
SV	2,348	0.04	1, 16	0.68	0.42	0.07	1, 16	1.16	0.30
WD_I	1,802	0.02	1, 16	0.32	0.58	0.01	1, 16	0.11	0.75
WD_SR	2,041	0.11	1, 16	1.90	0.19	0.10	1, 16	1.73	0.21
STATEWIDE	2,123	0.08	1, 16	1.37	0.26	0.00	1, 16	0.06	0.81
